# Advancing CART therapy for acute myeloid leukemia: recent breakthroughs and strategies for future development

**DOI:** 10.3389/fimmu.2023.1260470

**Published:** 2023-11-30

**Authors:** Lorena Pérez-Amill, Àlex Bataller, Julio Delgado, Jordi Esteve, Manel Juan, Nela Klein-González

**Affiliations:** ^1^ Fundació de Recerca Clínic Barcelona-Institut d’Investigacions Biomèdiques August Pi i Sunyer, Barcelona, Spain; ^2^ Gyala Therapeutics S.L, Barcelona, Spain; ^3^ Department of Immunology, Centre de Diagnòstic Biomèdic (CDB), Hospital Clínic de Barcelona, Barcelona, Spain; ^4^ Department of Haematology, Institut Clínic de Malalties Hematològiques i Oncològiques (ICHMO), Hospital Clínic de Barcelona, Barcelona, Spain; ^5^ Josep Carreras Leukemia Research Institute, Department of Biomedicine, School of Medicine, University of Barcelona, Barcelona, Spain; ^6^ Universitat de Barcelona, Barcelona, Spain; ^7^ Hospital Sant Joan de Déu, Universidad de Barcelona, Barcelona, Spain

**Keywords:** acute myeloid leukemia, chimeric antigen receptor, myelotoxicity, hematologic toxicity, cytopenia, on-target/off-tumor toxicity

## Abstract

Chimeric antigen receptor (CAR) T therapies are being developed for acute myeloid leukemia (AML) on the basis of the results obtained for other haematological malignancies and the need of new treatments for relapsed and refractory AML. The biggest challenge of CART therapy for AML is to identify a specific target antigen, since antigens expressed in AML cells are usually shared with healthy haematopoietic stem cells (HSC). The concomitant expression of the target antigen on both tumour and HSC may lead to on-target/off-tumour toxicity. In this review, we guide researchers to design, develop, and translate to the clinic CART therapies for the treatment of AML. Specifically, we describe what issues have to be considered to design these therapies; what *in vitro* and *in vivo* assays can be used to prove their efficacy and safety; and what expertise and facilities are needed to treat and manage patients at the hospital.

## 1 Introduction

### 1.1 History of cancer immunotherapy

The beginning of cancer immunotherapy can be traced back to William Coley, often hailed as the pioneer of this field. In 1891, Coley embarked on a groundbreaking endeavour to stimulate the immune system as a means to treat sarcoma patients. This involved injecting heat-inactivated *Streptococcus pyogenes* and *Serratia marcescens* to these patients (1). However, it was not until the last century that several breakthroughs in immunotherapy, including the development of monoclonal antibodies, the utilization of cytokines, oncology vaccines, and the introduction of immune checkpoint inhibitors (such as CTLA-4 and PD-1) (2), along with the adoptive cell therapy, catapulted immunotherapy into becoming the most promising approach for cancer treatment.

The first foray into cell immunotherapy against cancer occurred with the introduction of allogeneic stem cell transplantation in 1957 by E. Donall Thomas. Leukaemia patients were treated with intravenous infusion of bone marrow from healthy donors, inducing the “graft-versus-leukemia” effect (3, 4). A significant stride in adoptive cell therapy (ACT) occurred in 1986 when Dr. Rosenberg and his team described the use of tumour infiltrating lymphocytes (TILS) from melanoma patients. These TILS were isolated from melanoma surgical specimens, expanded *in vitro* with IL-2 for several weeks, and subsequently reintroduced into melanoma patient, resulting substantial tumour regressions (5).

In subsequent years, two different groups (Kuwana Y, et al. from Japan and Gross G, et al. from Israel) described the concept of covalently linking the antibody’s variable domains (V_L_, V_H_) to the TCR constant regions (C_α_, C_β_), thereby activating T cells in an HLA-independent manner (6, 7). Subsequently, Eshhar et al. and Brocker T, et al. independently designed a construct composed of a single-chain variable fragment (scFv) of an antibody linked to the signalling ζ or γ chain of the T-cell receptor (TCR), allowing the entire expression in one molecule, thus generating the first CAR molecule (8–11). Since this initial CAR design, numerous generations of CAR molecules have been developed (section 1.2).

**Figure 1:**
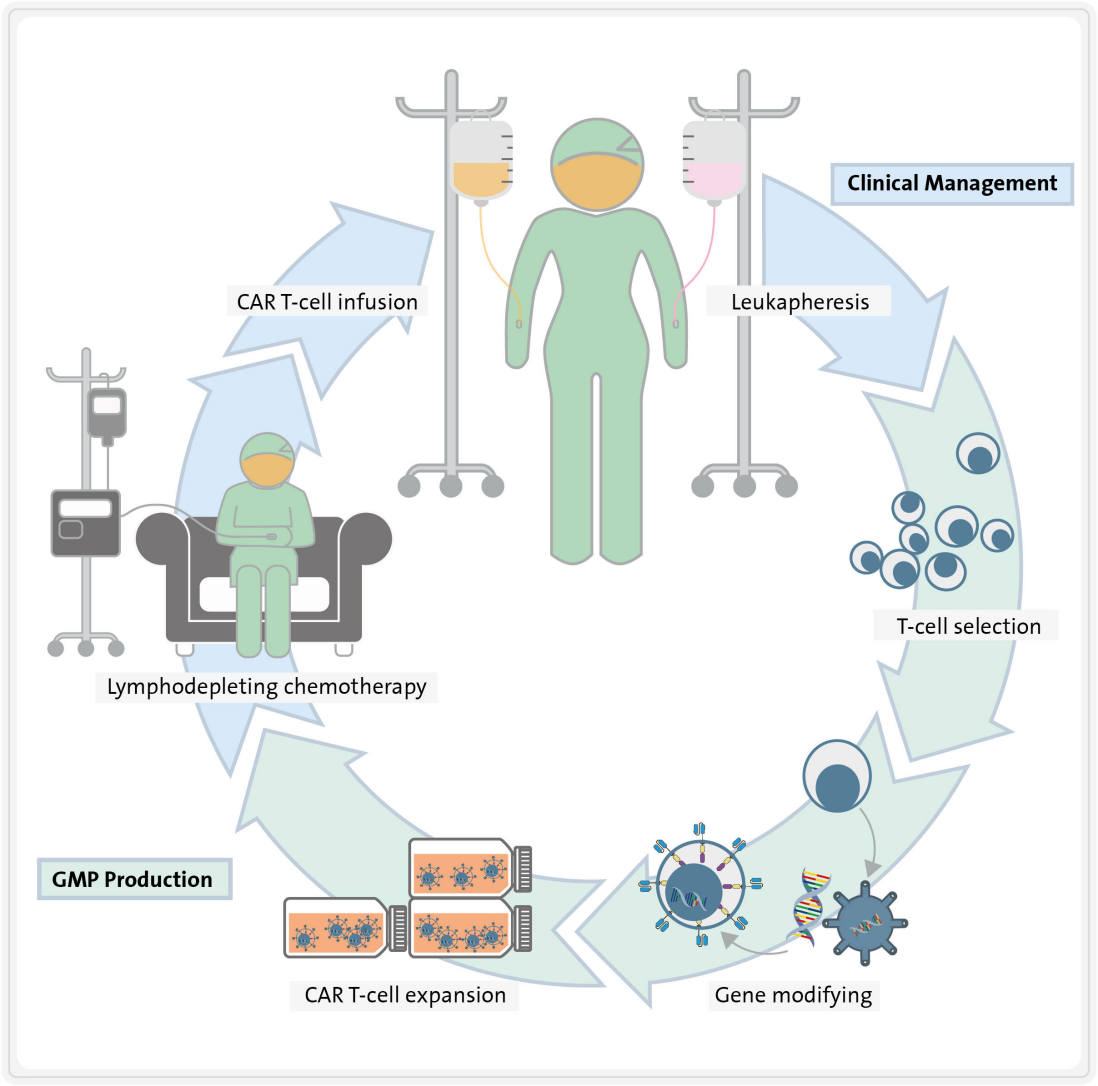
Steps required for CART therapy. First, T cells are isolated from the patient blood by leukapheresis. Second, they are activated and genetically engineered *ex vivo* to express the CARs on their surface. Third, they are expanded in close-manufacturing bioreactors, while patient undergoes lymphodepleting chemotherapy. Finally, CART cells are re-infused into the patient, where they exert specific cytotoxicity towards tumour cells.

Today, CAR molecules are synthetic chimeric receptors comprising an extracellular antigen-binding domain derived from an antibody and the intracellular signalling domain of the TCR. CART therapy involves modifying T cells to express CARs that recognize a specific antigen expressed on the surface of malignant cells and exert a cytotoxic effect towards them in an HLA-independent manner. Autologous T cells are isolated from the patient’s blood by leukapheresis, activated, genetically engineered *ex vivo* to express the CAR on their surface, and expanded in close-manufacturing bioreactors. After obtaining and characterizing the cell product, these cells are frequently cryopreserved. To ensure the engraftment of CART cells, patients usually undergo a lymphodepleting chemotherapy prior to CART infusion (**Figure 1**). Finally, they are infused into the patient to specifically kill cancer cells expressing the target antigen (**Figure 1**) (12, 13).

### 1.2 CAR structure

**Figure 2:**
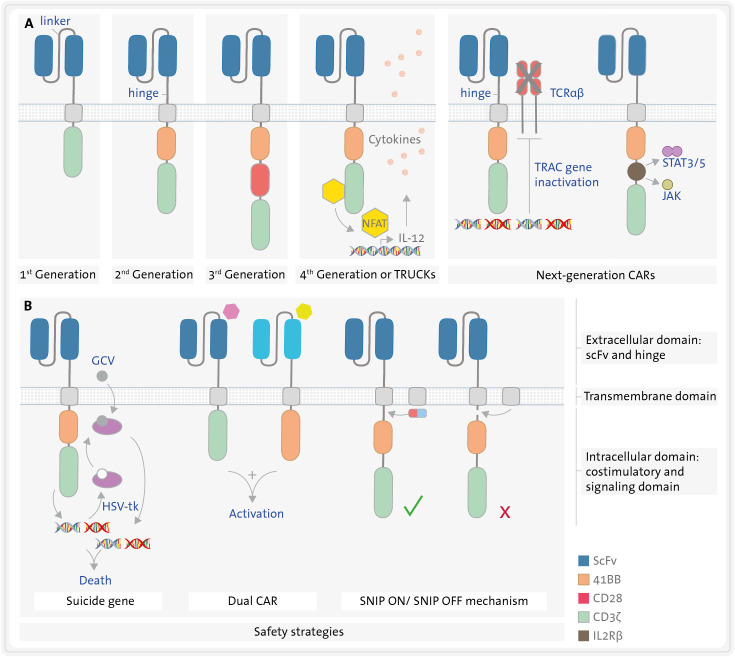
Structure of different CAR constructs. **(A)** CARs have three domains: an extracellular, a transmembrane, and an intracellular domain. The extracellular domain (blue) consists of a single chain variable fragment (scFv) formed by the variable region of a heavy chain (VH) and that of a light chain (VL) derived from an immunoglobulin, both connected by a linker. A hinge connects the extracellular domain to the transmembrane domain (grey). The intracellular domain consists of a signalling domain (green) and several possible costimulatory domains (orange, red, yellow, brown), such as 4-1BB or CD28. Five generations of CART cells varying in the number and type of costimulatory domains are represented in section **(A, B)**. Safety strategies **(B)** include the use of suicide genes e.g., herpes virus thymidine kinase (HSV-tk) in combination with ganciclovir (GCV), dual CART cells, and inducible CART cells using SNIP mechanism. JAK/STAT3/5, janus kinase-signal transducer and activator of transcription pathway; NFAT, nuclear factor of activated T cells; SNIP, Signal Neutralization by an Inhibitable Protease; TRAC, T-cell receptor alpha constant; TRUCKs, T cells Redirected for Universal Cytokine Killing.

CAR molecules are synthetic chimeric receptors characterized by three distinct domains: the extracellular antigen-binding domain, the transmembrane domain, and the intracellular CD3ζ signalling chain of the TCR (11, 14, 15) (**Figure 2**).

The extracellular domain is composed of a scFv. This is the domain that recognizes the antigen expressed on the surface of target cells. The scFv is a fusion protein that combined the variable region of a the heavy (VH) and the variable region of the light chain (VL) of an immunoglobulin, fused by a linker that confers its flexibility (16). Typically, it is derived from a murine antibody, although it can also be humanized or even fully human (e.g. originating from transgenic mice or a phage-display library) (17). The latter options may be preferred in certain circumstances over murine scFv as the latter can potentially lead to immunogenicity. However, disparate data have been reported about the immunogenicity induced by CART therapy (18–20). Some patients may develop a humoral anti-CAR response, the so-called HAMAs (human anti-mouse antibody) (21), which have been reported to potentially reduce the persistence and cytotoxic effect of the CART cells (22, 23). Nevertheless, other groups using the commercial CART anti-CD19 tisagenlecleucel did not observe any reduction in CART expansion, persistence or efficacy due to HAMAs (24).

The spacer or hinge connects the extracellular and the transmembrane domains, providing flexibility to the scFv and enhancing the interaction between the CAR and the target cell (25). Additionally, its length can influence the functionality of the immunological synapse (26). The transmembrane domain is embedded in the cellular membrane linking the extracellular and intracellular domains of the CAR. Both the hinge and the transmembrane domain are typically derived from either CD28, CD8 (T-cell surface glycoproteins) or Immunoglobulin G (IgG) (27).

Depending on the intracellular domain employed, in accordance with their evolutionary trajectory, five generations of CARs can be discerned, (**Figure 2**). The intracellular CD3ζ chain from the TCR complex (signalling domain) is a common feature across all CAR versions. It initiates the first signal for T-cell activation (22, 28). First-generation CARs rely only on this domain to promote the activation of CART cells, which does not ensure sustained cytotoxic activity *in vivo*. Consequently, second and third generations of CARs were engineered with one or two additional costimulatory domains, respectively, incorporating the second costimulatory signal requisite for T lymphocyte activation. These augment activation levels, efficacy against target cells and persistence of the CART cells *in vivo* (29, 30) (**Figure 2**). The most frequently employed costimulatory domains are CD28 (31) and 4-1BB (CD137) (32), although others like ICOS, OX40, CD27 or DAP-12 have also been utilized (15). Clinical findings indicate that CARs with a CD28 domain lead to greater T-cell activation, while those with a 4-1BB domain result in enhanced persistence *in vivo* (33–35).

Fourth-generation CARs also produce and secrete transgenic proteins, such us cytokines like interleukin 12 (IL-12) under the control of the nuclear factor of activated T cells (NFAT) (15, 36). Cytokine secretion can either improve the activation of CART cells or diminish the immunosuppressive tumour microenvironment, thereby increasing their survival and persistence. This, in turn, translates into a higher cytotoxicity over an extended period in preclinical models (37). Fourth-generation CARs are also referred to TRUCKs: T cells Redirected for Universal Cytokine Killing (37).

Finally, consensus is lacking regarding what constitutes a “fifth-generation”. In fact, next-generation CART cells come in at least two flavours. The first option involves a signal from a truncated cytoplasmatic cytokine receptor, such as IL2Rβ, which activates the JAK-STAT3/5 pathway. These CART cells have three immune activation signals – TCR activation by the CD3ζ, the costimulatory signal (typically 4-1BB or CD28) and the cytokine signal through IL2Rβ (38). The second option entails gene-edited CART cells, for instance, with inactivation of the T-cell receptor alpha constant (TRAC) gene via CRISPR-Cas9, to prevent TCR expression for donor-derived allogeneic CART cells (39) (**Figure 2A**).

CAR construct modifications focusing on safety will be discussed in more detail later. Briefly, there are several strategies that employ a suicide gene or express an antigen on the CART cells. This allows for the targeting of CART cells by approved antibodies, inducing antibody-dependent cell-mediated cytotoxicity (ADCC) and enabling the elimination of CART cells in cases of severe toxicity (40) (**Figure 2B**). Other strategies to enhance CART safety involve engineering them to exert a cytotoxic effect only when more than one target antigen is detected (e.g., dual CART cells) or conditionally expressing CAR molecules, such as Signal Neutralization by an Inhibitable Protease (SNIP) (41) (**Figure 2B**).

### 1.3 Clinical approved CART products

The initial successful clinical outcomes of CART cells were simultaneously reported by three distinct institutions: 1) the National Cancer Institute (NCI) (42), 2) the Memorial Sloan-Kettering Cancer Center (MSKCC) (43) and 3) the University of Pennsylvania (UPenn) (44). Various second-generation CART therapies targeting the CD19 antigen were developed and assessed in patients. The NCI group treated a patient with advanced follicular lymphoma, resulting in a partial response and B-cell aplasia after CART treatment (42). At MSKCC Hospital, nine patients diagnosed with refractory chronic lymphocytic leukemia (CLL) or relapsed B-cell acute lymphoblastic leukemia (B-ALL) were treated with CART therapy, demonstrating both safety and promising therapeutic potential (43). Meanwhile, UPenn reported the initial complete response of patient with refractory CLL treated with anti-CD19 CART therapy (44).

**Figure 3:**
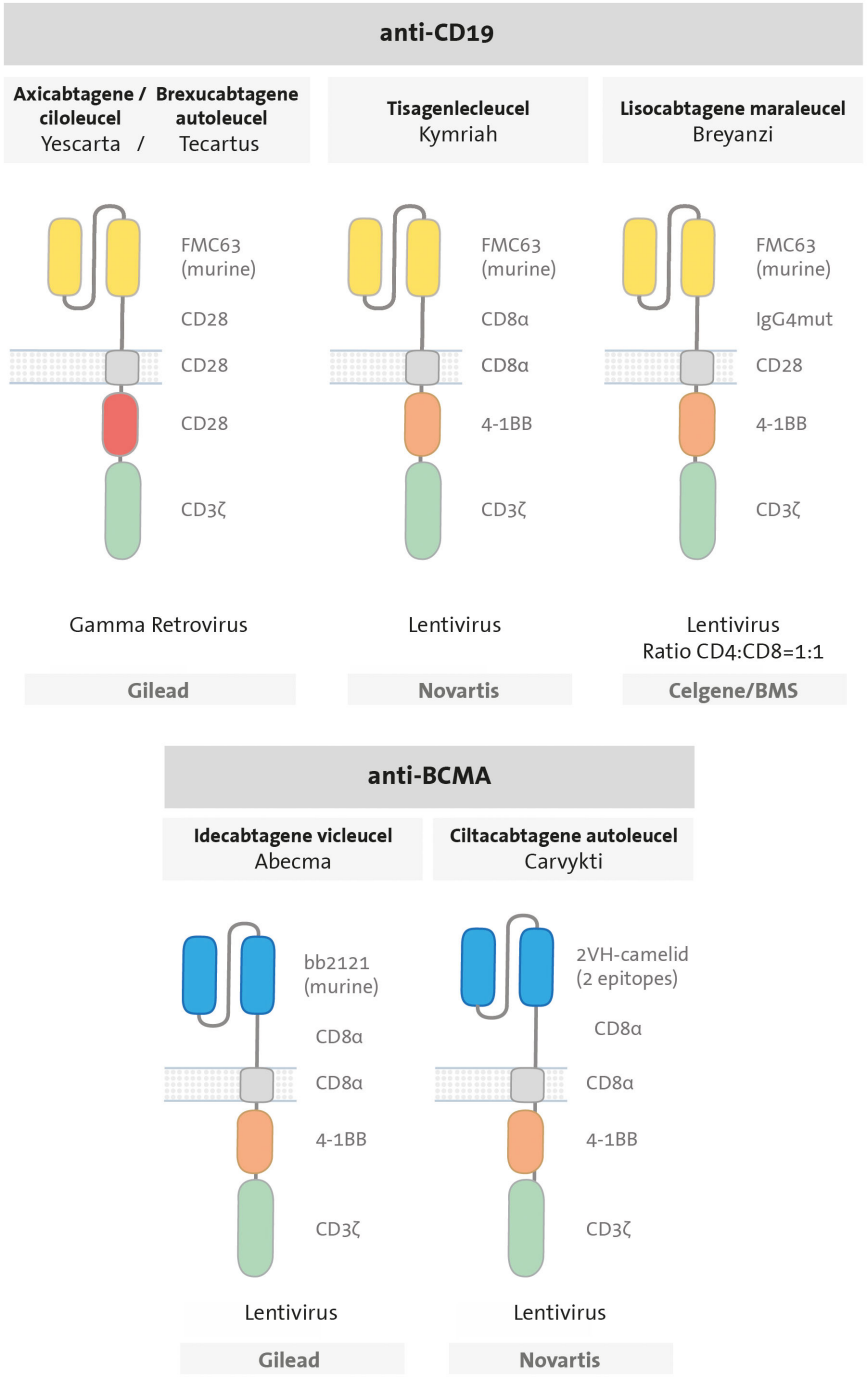
Design of CAR T cells approved by the FDA. All are second-generation CARs, composed by an scFv (anti-CD19 in yellow or anti-BCMA in blue); a hinge (CD28, CD8a or IgG4mut); a transmembrane domain (CD28 or CD8 in grey), one costimulatory domain (CD28 in red or 4-1BB in orange) and CD3ζ signalling domain in green.

Since then, six CART therapies have received approval from the U.S. Food and Drug Administration (FDA) (**Figure 3**). Among them, four of them are CART cells targeting the B-cell antigen CD19: tisagenlecleucel (Kymriah) (45), axicabtagene ciloleucel (Yescarta) (46), brexucabtagene autoleucel (Tecartus) (47), and lisocabtagene maraleucel (Breyanzi) (48). The remaining two target the B-cell maturation antigen (BCMA): idecabtagene vicleucel (Abecma) (49) and ciltacabtagene autoleucel (Carvykti) (50). CD19-directed CART therapies are approved for treatment of patients with relapsed or refractory (R/R) B-ALL and B-cell lymphomas; while BCMA-directed CART therapies are indicated for R/R multiple myeloma (MM). All approved CART products express second-generation CARs, with either a 4-1BB or CD28 costimulatory domain. The majority of approved products employ a murine scFv with the exception of ciltacabtagene autoleucel, which utilizes a 2-epitope binding camelid. The different domains utilized in each CAR, including the hinge and TM domains, are illustrated in **Figure 3**. Additionally, there is the option of clinical application via a clause known as Hospital Exemption, as exemplified by varnimcabtagene autoleucel for the treatment of adult patients with R/R ALL in Spain (51).

## 2 Design of CART therapy for AML

### 2.1 State of the art of AML treatment

Acute myeloid leukemia (AML) is a heterogeneous neoplasm characterized by uncontrolled clonal expansion of transformed immature haematopoietic precursors, often associated with recurrent genetic alterations (52, 53). Its incidence is approximately 4.2 cases per 100,000 habitants, with a median age of presentation of 68 years, although it can be manifest at any age (54, 55).

For patients eligible for intensive treatments, chemotherapeutic agents remain the therapy of choice, typically including cytarabine and anthracyclines in most cases. However, new drugs are progressively being introduced. For patients who are not eligible for intensive treatments, novel active low-intensity regimens have been emerged and provide meaningful antileukemic activity. These include the combination of the bcl-2 inhibitor, venetoclax, with hypomethylating agents or low-dose cytarabine, as well as the isocitrate dehydrogenases 1 (IDH1) inhibitor, ivosidenib, for AML cases harbouring an activating mutation in this metabolic gene (56, 57). Nonetheless, none of these therapeutic approaches lead to complete leukemia eradication, and most patients will experience a clinical progression after several cycles.

Allogeneic haematopoietic stem-cell transplantation (allo-HSCT) has been a pivotal procedure for non-favourable risk AML, due to its potential to eliminate residual leukemic cells through the graft-versus-leukemia (GvL) effect (58, 59). Relapsed or refractory (R/R) AML presents a frequent and challenging scenario, occurring in 40-50% of younger patients and even more frequently in older individuals. A standard treatment protocol for these patients is still lacking and survival is poor, with an overall survival rate of 10% at 5 years (60, 61). Therefore, there is a clear need for novel therapeutic approaches in this scenario.

The therapeutic landscape of AML has undergone recent transformation with the introduction of monoclonal antibodies (mAb), such as CD33 gemtuzumab ozogamicin, (GO) and targeted therapies like the aforementioned venetoclax, as well as IDH1/2 and FLT3 inhibitors (56, 57, 62). Novel immunotherapeutic approaches are also being explored. For instance, ongoing clinical trials are assessing the efficacy of CD3-engaging bispecific antibodies, such as the CD123xCD3-targeting flotetuzumab. Additional targets being explored include CD33xCD3 and WT1xCD3 (63, 64).

Another interesting approach involves utilizing drugs relieving the blockage imposed by various immune checkpoints expressed by leukemic cells. Whereas CTLA-4 and PD-1 inhibitors, which are efficacious in several solid tumours and specific lymphoma subtypes, have not exhibited significant antileukemic activity in AML, promising initial results have been reported targeting other innate immune checkpoints. For example, the anti-TIM3 monoclonal antibody sabatolimab or the antibodies magrolimab and lemzoparlimab targeting CD47 (“do not eat me” signal), may restore macrophage phagocytosis of AML cells, and are currently under investigation in several clinical trials (65–67).

While several CART products targeting CD19 or BCMA have yielded outstanding clinical outcomes and some have been gained regulatory approval for treating B-cell malignancies, none has yet received regulatory approval for AML. In the subsequent sections, we describe the CART approaches that have been tested or are currently under investigation in AML. Finally, we integrate published data with the expertise of our group in the development CART products, offering guidance to researchers on how to design, develop, and translate these therapies for the treatment of AML.

### 2.2 Designing of CART for AML

The foremost challenge in CART therapy for AML lies in identifying a specific target antigen that is expressed on the surface of malignant cells but not on healthy cells, since the concomitant expression of the target antigen on both leukemic and healthy cells could result in on-target/off-tumour toxicity of varying severity. For instance, in B-cell malignancies, CD19-directed CART cells eradicate both malignant and healthy B cells/B-cell progenitors. However, B-cell aplasia and consequent hypogammaglobulinemia can be effectively managed with intravenous immunoglobulin reposition (68). In contrast, in some cases, on-target/off-tumour toxicity can be fatal, as was the case of a patient who died five days after receiving ERBB2-directed CART therapy for metastatic colon cancer. In this instance, CART cells targeted pulmonary epithelial cells expressing the ERBB2 antigen leading to severe respiratory distress followed by cardiac arrest (69). Therefore, choosing an appropriate target is the pivotal initial step in designing a CART cell (70).

The majority of surface antigens identified to date in AML cells are either shared with healthy haematopoietic stem cells (HSC) or not universally expressed in all AML cells. The on-target/off-tumour effect of CART cells on HSC could result in prolonged cytopenia, putting the patient at risk of infections or bleeding. This challenge can only be circumvented if targets exclusively expressed in AML cells are identified. This can be achieved, for example, through whole-genome sequencing (71), scrutinizing the surfaceome of AML (72) or carrying out proteomic and transcriptomic studies to compare antigen expression in leukemic stem cells and healthy stem cells (70). The ideal target antigen should possess the following characteristics: 1) restricted expression to malignant cells, i.e. minimally or not expressed at all on their healthy counterparts to minimize hematologic toxicity; 2) restricted expression to malignant cells without being expressed on other healthy tissues to avoid further on-target/off-tumour toxicity; 3) prevalent expression in most AML cases, enduring over time and under selective pressure; 4) expression in both malignant myeloid mature blasts and leukemic stem cells (LSC) to prevent the escape of the latter, which could lead to relapse (73).

### 2.3 Common antigens targeted by CART in AML

**Figure 4:**
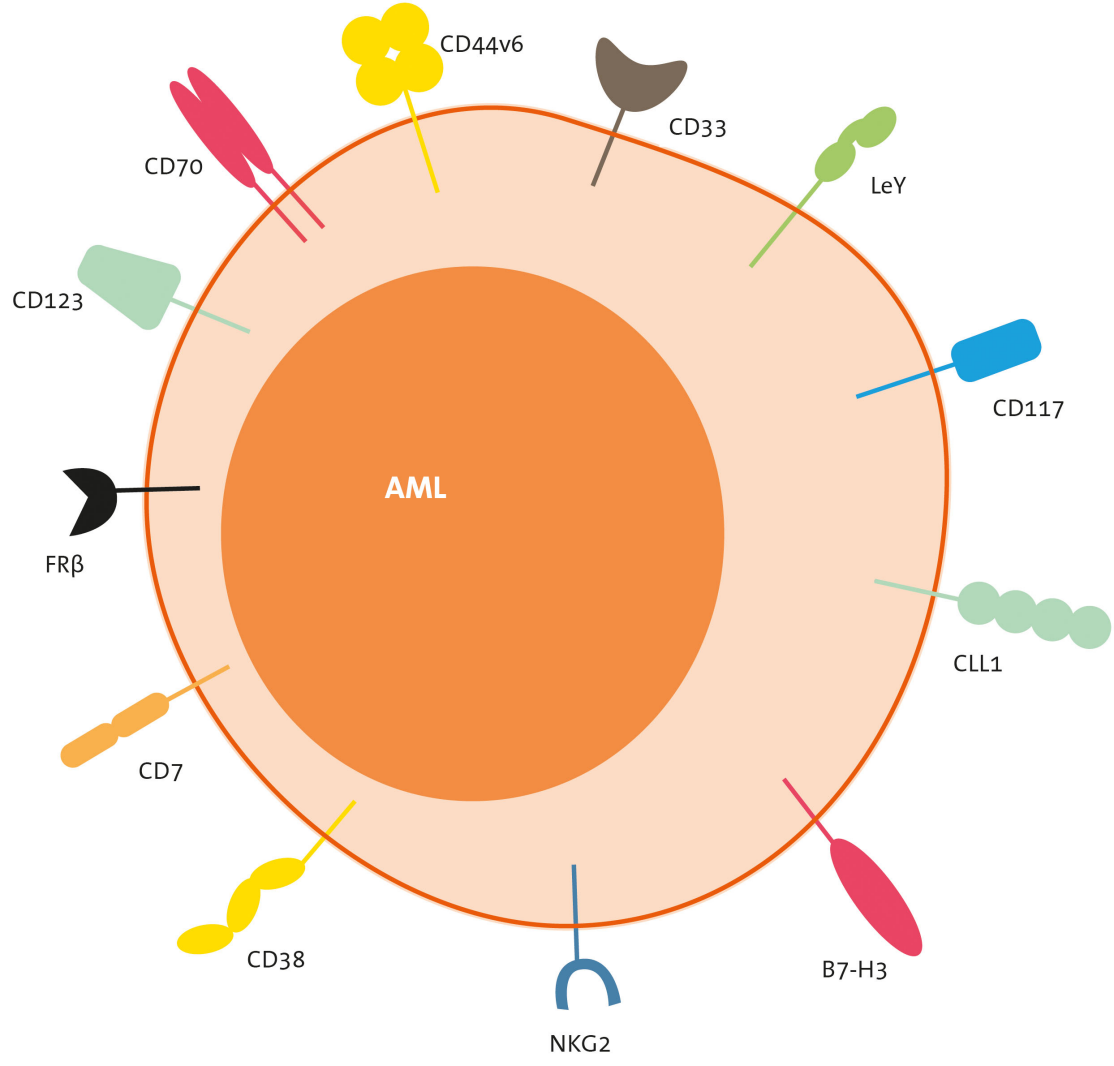
Target antigens of CART therapies for AML. CLL-1, C-type lectin-like molecule-1; FLT-3, FMS-like tyrosine kinase 3; FRb, folate receptor b; LeY, Lewis Y; NKG2, natural killer group 2 member.

In this section, we will discuss the most frequently targeted antigens by CART therapies (**Figure 4**), and later, we will provide a summary of the pertinent clinical data associated with these products (**Table 1**).

**Table 1:** Published clinical trials using CART in AML: CART design.

Clinical trial N°	Target	Vector	ScFv	Construct	Cell source	Reference
NCT01716364	Lewis Y	Retroviral	Humanized	LeY-CD8αH-CD28TM-CD28-CD3ζ	Autologous	(74)
NCT02623582	CD123	mRNA	NA	mRNA (biodegradable) CD123-41BB	Autologous	(75)
NCT02159495	CD123	Lentiviral	Murine	CD123-IgG4op-CD28-CD3ζ, safety switch EGFRt	Autologous	(76)
NCT04318678	CD123	Lentiviral	NA	CD123-CD28-CD3ζ, safety switch CD20	Autologous	(77)
NCT04106076	CD123	Lentiviral	Murine	universal CD123-CD8HTM-41BB-CD3ζ, safety switch RQR8, TCR KO, CD52 KO	Allogeneic	(78)
NCT01864902	CD33	Lentiviral	NA	CD33-CD8αHTM−41BB-CD3ζ	Autologous	(79)
NCT03927261	CD33	Non-viral gene transfer	NA	CD33-mbIL15, safety switch	Autologous	(80)
NCT03795779	CD33-CLL1	NA	NA	CLL1-P2A-CD33 intracellular part NA	Autologous	(81)
NA	CLL1	NA	Murine	NA	Autologous	(82)
NCT03222674/ ChiCTR1800015883	CLL1	Lentiviral	Humanized	4th generation CLL1-CD28-CD27-CD3ζ, safety switch iCasp9	Autologous	(83)
ChiCTR2000041054	CLL1	NA	NA	NA	Autologous	(84)
NCT03222674/ ChiCTR1900027684	CCL1	Lentiviral	Murine	CLL1-41BB-CD3ζ CART	Autologous	(85)
NCT02203825	NKG2D ligands	Retroviral	Human	NKG2D-CD3ζ	Autologous	(86)
NCT04351022	CD38	NA	NA	3rd generation CD38-CD8αHTM-CD28-41BB-CD3ζ	Autologous/Allogeneic	(87)
NCT04538599	CD7	Retroviral	NA	universal CD7-CD8αHTM-41BB-CD3ζ, CD7 KO, TCR KO, HLA−II KO, NK inhibitory receptor	Allogeneic	(88)

H, hinge; iCasp9, inducible caspase 9 motif; TM, transmembrane domain; NA, not available.

CD33 is a transmembrane receptor expressed in the majority of AML cases (approximately 80%) and has therefore been extensively studied as a target for CART therapy in AML (89, 90). However, it is also expressed on myeloid cells, both mature and progenitors, as well as on certain cells of lymphoid lineage (91, 92). The humanized anti-CD33 antibody drug-conjugated GO (GO, Mylotarg® Pfizer) has gained approval for frontline therapy, in combination with intensive chemotherapy, especially in cases with favourable-risk cytogenetics AML (93). The main toxicities associated with GO include myelotoxity and the risk of inducing sinusoidal obstruction syndrome (SOS). Nonetheless, this latter side effect appears to be target-independent damage, probably related to the conjugated moiety of the drug, calicheamicin. This is evident as a similar toxicity is also observed with inotuzumab ozogamicin, an anti-CD22 antibody conjugated with the same molecule (94, 95).

CD123 or alpha chain of the interleukin 3 receptor (IL-3Rα) is expressed in 80% to 90% of AML cases. Moreover, it is prevalent not only in the bulk of AML blasts but also in LSC. Preclinical studies have demonstrated robust anti-leukemia effects with CD123-directed CART cells (96–98). Several CART therapies targeting this antigen are currently in clinical development for AML. However, this antigen is also expressed on healthy myeloid lineage cells, and there is conflicting data regarding the myelotoxic effect of CD123-directed CART cells. Some studies report low expression of CD123 on HSC (99, 100) while others describe a myeloablative effect of CD123-directed CART cells in various humanized mouse models (101). Consequently, in most clinical trials, CD123-directed CART therapy is utilized as a bridge therapy to allo-HSCT (see section on Clinical trials in AML) (76).

Additionally, CD123 is expressed on healthy endothelial cells of small-calibre blood vessels (102, 103) and this introduces another potential on-target/off-tumour effect of CD123-directed CART cells, namely capillary leak syndrome. CD123 has been clinically validated as a target for the treatment of blastic plasmacytoid dendritic-cell neoplasm (BPDCN) with the use of tagraxofusp (SL-401), a CD123-directed cytotoxin containing a truncated diphtheria toxin (70). Notably, capillary leak syndrome was a frequent adverse effect experienced by approximately 25% of patients.

CLL-1 or C-type lectin-like molecule 1 (also known as C-type lectin domain family 12 member A, CLEC12A) is expressed in over 80% of AML cases, on both blasts and LSC (104, 105). It is also expressed on healthy differentiated myeloid cells, but notably not on HSC or other non-haematologic human tissues (104, 106). Preclinical studies have demonstrated robust anti-leukemia activity of CLL-1-directed CART cells, both *in vitro* and in *in vivo*, without causing myelosuppression (107–109). This makes CLL-1 a promising target for CART therapy in AML.

In addition to the previously mentioned targets, which are common myeloid antigens, several others are currently under exploration. For instance, NKG2D exhibits an up-regulation in AML and while maintaining limited expression in healthy tissues. Nevertheless, it is important to note that inflammation and stressful events can lead to an up-regulation of this antigen in healthy tissues (110). CD7 is expressed in approximately 30% of adult AML cases and is associated with a more aggressive course of disease. However, it is also expressed on normal activated T cells, NK cells, and some progenitor cells. Consequently, CD7-directed CART therapies may lead to CART fratricide, and knocking out CD7 in autologous cells might be a necessary step in the CART generation process.

Other potential targets include Lewis Y (LeY), which was the initial targeted in CART therapy for AML patients (69, 70); FLT3 (111, 112); folate receptor β (113, 114); CD38 (115); CD44v6 (116, 117); CD117 or c-kit (118); CD276 (119); and B7-H3 (120). All these targets have shown promising preclinical results.

### 2.4 Future prospects of CART in AML

While various antigens have been explored in preclinical and clinical settings for CART in AML, none has yet demonstrated results comparable to those achieved with CD19 antigens in the context of B-cell malignancies, both in terms of efficacy and safety. However, there are emerging strategies that hold the potential to broaden the therapeutic window of CART therapy in AML.

#### 2.4.1 Strategies to enhance efficacy

The choice of various domains in CART cells is pivotal in increasing their efficacy and persistence. The scFv derived from different murine antibodies, humanized antibodies or fully human scFv exhibit varying binding affinities to antigens (121). Hence, it proves beneficial to assess multiple scFv directly against the same antigen and select the one that best suits the chosen strategy. Additionally, studies have noted that CART cells containing CD28 as the costimulatory domain display greater sensitivity to low levels of antigen compared to CART with 4-1BB. On the other hand, CART cells containing the 4-1BB costimulatory domain have demonstrated increased persistence *in vitro, in vivo* and in clinical trials (35, 122, 123).

TRUCKs, the fourth generation of CART, offer two distinct avenues for enhancement. They can heighten the cytotoxicity of CART cells by releasing cytokines like IL-12, or alternatively, promote their expansion and persistence through the release of cytokines such as IL-15. Atilla PA and collaborators found that CLL1-CART cells co-expressing transgenic IL-15 displayed a less terminally differentiated state and demonstrated superior expansion compared to CART cells lacking IL-15 (124). Moving forward, the fifth generation of CART cells further heightens the persistence of CART cells through the provision of a third immune activation signal. In addition, gene editing of the CAR gene in *TRAC* locus using CRISPR-Cas9 technology presents another option to enhance the potency of CART cells (39).

The combination of CART therapy with other immunotherapies, such as immune checkpoints inhibitors, holds the potential for a synergistic effect, ultimately increasing its efficacy. Ongoing clinical trials are investigating whether the combination of CART-19 and PD-1 or PD-L1 blockade can enhance CART persistence and clinical responses (125, 126). Given the heterogeneity of AML, selecting a single antigen present in all tumoral cells can be challenging. Some identified antigens for AML exhibit a regulatable expression. For instance, CD70 is widely expressed on AML cells but not on normal HSC and its expression on malignant cells can be heightened by the use of azacytidine (127). Similarly, the folate receptor B is typically expressed on 70% of AML cells, but its expression can be upregulated when AML cells are treated with all-trans retinoic acid (ATRA) (113).

An alternative approach involves engineering dual CART cells to modulate antigen recognition on tumour cells. There exists a wide range of dual CAR options, employing Boolean logic to determine when CART cells will be activated. Three primary logic gates can be distinguished: 1) AND: both antigens must be recognized for CART activation; 2) OR: either one of the two antigens must be recognized for CART activation, and 3) NOT: only antigen 1 must be present; the presence of antigen 2 inhibits CART activation (128). In cases where the antigen is downregulated or expressed at low levels, a dual CAR with an “OR logic-gate” strategy may prevent tumour escape. Here, CART is fully activated when targeting either one of the two target antigens (e.g. tandem, bicistronic or co-infusion CARTs).

Taking this concept further, a combination of a CAR with an HLA-Independent TCR (HIT) can increase sensitivity and decrease tumour escape associated with low target antigen expression. Theoretically, this strategy allows for a more precise selection of both target antigens compared to the dual strategy. Mansilla-soto and collaborators proposed choosing a lower-density target for the HIT (increasing sensitivity to this target and avoiding antigen escape) and a higher-density target for the CAR (129).

#### 2.4.2 Strategies to enhance safety

Currently, the majority of procedures in clinical trials involving CART cells in AML are followed by an allo-HSCT to overcome their potential myelotoxic effect. One strategy to improve safety involves engineering dual CART cells that calibrate their affinity to antigens present on both malignant and healthy cells, thereby reducing the on-target/off-tumour effect.

One approach is to design dual CART cells that only eliminate cells that express both target antigens, utilizing an “AND logic-gate” strategy, as previously described (**Figure 2**). In this scenario, two CAR molecules are expressed in the same T cell. One CAR molecule is a first-generation CAR that recognizes antigen 1 with the intracellular CD3ζ signalling domain and the second CAR molecule is a chimeric costimulatory receptor (CCR) that recognizes antigen 2 with an intracellular costimulatory domain (130). With this AND-gate strategy, CART cells are fully activated only when encountering both antigens (131–133). For instance, dual CART cells can be engineered to target a specific leukemia antigen (e.g., CD7) and an antigen both expressed on the leukemic blasts and on HSC (e.g., CD33). These bispecific CART cells would exert a cytotoxic effect only when encountering leukemic cells expressing both target antigens (i.e., CD33+ CD7+), but not when encountering HSC (CD33+ CD7-) or mature T cells (CD33-, CD7+) (134).

Another innovative approach is the new “IF-better gate” strategy, which modulates the detection and activity of CART cells based on the density of antigens expressed on both leukemic cells and healthy cells. In this scenario, the CART cell targets malignant cells with a high density of target 1 through the CAR (first construct). Cells with a low density of target 1 are only eliminated when they also exhibit a high density of target 2, recognized simultaneously by the concomitant CCR recognition (135, 136).

Another approach is the “NOT logic-gate” strategy. In this scenario, the first CAR molecule recognizes an antigen present on tumour cells, while the second CAR recognizes an antigen present only on healthy cells. This second recognition inhibits the activation of the first CAR in a reversible manner (130).

Utilizing mRNA CART cells is a different strategy to enhance safety; mRNA CART are “biodegradable” CARs since RNA is not integrated into the genome. This means they will not persist in the patient for more than a few days, aiming to mitigate myelotoxicity. However, this approach carries the potential risk of disease relapse due to low persistence of CART cells (137, 138). This mRNA strategy has been studied both *in vitro* and *in vivo* with CD33 (139) and CD123 (140) antigens for AML. Nevertheless, initial clinical trials with CD123 “biodegradable” CART cells did not yield clinical responses (75).

To mitigate myelotoxicity, one approach is to generate CART cells targeting an antigen on leukemic cells that has been deliberately knocked out from the donor HSC for the subsequent allo-HSCT (141). Kim and co-workers successfully deleted CD33 from HSC, leading to a long-term and functional engraftment with an immune system resistant to the anti-CD33 CART cells in a xenograft mouse model (141). Similarly, Nils and colleagues published a CART therapy targeting the pan-haematologic CD45 antigen. They base-edited the targeted CD45 epitope in human HSC (CD45^edited^) to prevent their killing by the anti-CD45 CART cells (142, 143).

Another alternative to mitigate both myelosuppression and cytokine release syndrome (CRS) induced by CART cells is the implementation of switch-off strategies (**Figure 2**) (40, 133), such as the expression of suicide genes. For instance, the inducible Caspase-9 suicide gene (iCasp9) can be expressed in CART cells, where the administration of the AP1903 molecule induces the Caspase3 apoptosis pathway in this cell. Another well-studied suicide gene is the Herpes Simplex virus thymidine kinase (HSV-tk); with the addition of ganciclovir (GCV); the HSV-tk phosphorylates GCV developing a toxic triphosphate which competes with triphosphate triggering to DNA synthesis inhibition and cell apoptosis (144). The other switch-off strategy, as previously described, is to express an antigen on the CART cells that can be targeted by antibodies to induce ADCC. For instance, the expression of EGFR or CD20 on the surface of CART cells, that could be targeted with the monoclonal antibodies cetuximab or rituximab, respectively, to induce ADCC and eliminate the CART cells if needed (145). Moreover, recently, SNIP CART cells have been successfully engineered to be non-constitutively active, but instead switched between an ON- and an OFF-status by a protease, thus enabling tuning of CAR activity to improve safety (41, 146).

In conclusion, a range of strategies have been devised to enhance the safety of CART therapy in AML. These include the precise and sophisticated engineering of dual CART cells, the utilization of biodegradable mRNA CART cells, and the implementation of switch-off mechanisms. Additionally, gene editing of donor HSCs offer promising avenues to mitigate potential toxicities.

## 3 Preclinical development of new CART therapies for AML

### 3.1 Engineering CARTs

Creating effective CART therapies involves adjusting various components like the scFv’s affinity and recognized epitope. This includes the scFv itself, the order of its VH/VL domains, the length of the linker in between, as well as the hinge, TM domain, costimulatory domain/s, and implementing a safety strategy if wanted. By modifying one or more of these factors, different CARTs can be designed with distinct efficacy and safety profiles (147, 148). These CARTs are then assessed through a series of lab tests to select the most promising candidate for further clinical development.

### 3.2 Isolating, activating, and expanding effector cells

The majority of CART therapies currently in development utilize T cells, which are potent killers and relatively easy to manipulate. These T cells can be obtained either from the patient (autologous) or a healthy donor (allogeneic). By using autologous CART cells, the risk of graft-*versus*-host disease (GvHD) is avoided. In some cases, patients have relapsed after an allo-HSCT before receiving the CART treatment. In these cases, CART cells are engineered from autologous T cells, which technically come from the patient’s rebuilt donor immune system. Surprisingly, even in that case, significant GvHD has not been observed (149, 150). Alongside T cells, other immune cells like natural killer cells (NK), cytokine-induced killer cells (CIK), macrophages and regulatory or γδ T cells are being explored (151). NK or γδ T cells are advantageous as they do not cause GvHD, allowing them to be used in “off-the-shelf” allogeneic therapies (152–154). Another approach to prevent GvHD involves knocking out (KO) the endogenous T-cell receptor (TCR by *TRAC* gene KO) using genome editing tools like zing-finger nuclease or CRISPR/Cas9 (98, 154, 155).

From this point forward, we will focus on the steps needed to generate autologous CART cells. First, T cells are separated from peripheral blood mononuclear cells (PBMC) by isolating the cells that are positive for CD3 antigen. The ratio between the subpopulations of CD4^+^ helper T cells and CD8^+^ cytotoxic T cells can vary from person to person. Some studies suggest using products with a specific ratio (e.g., 1:1), while others do not adjust this parameter (48, 156). Currently, among the six FDA-approved CART products, only one has a defined 1:1 CD4:CD8 ratio (157). Further research is required to determine if a particular CD4:CD8 ratio is necessary to achieve better therapeutic results.

After isolating the T cells, they need to be activated with antibodies or antibody-coated beads (CD3-CD28) and cytokines (IL-2/IL-7/IL-15). Then, they are expanded using a culture medium supplemented with the cytokines (IL-2 or IL-7/IL-15) (158). Traditionally, IL-2 is used for *in vitro* expansion. However, it has been reported that using IL-15 leads to less T-cell senescence, which could improve their efficacy and persistence in the body (159).

### 3.3 Gene-editing

Gene editing can be achieved in two ways: permanently using through viral means (such as γ-retroviruses, lentiviruses, adenoviruses or adeno-associated viruses) or temporarily through non-viral means (like transposons or mRNA).

The retroviridae family includes γ-retroviruses and lentiviruses. They facilitate the integration of the new genetic information (transgene) into the genome of T cells, allowing for stable, long-term gene expression (112). While γ-retroviruses can only infect dividing-cells, lentiviruses can infect both dividing and non-dividing cells. Among the lentiviruses, the second and third generations lentiviruses (LV) are commonly used in this process. The former has a higher transduction rate, while the latter is considered safer (160). It is important to note that when using these methods, the transgene is inserted randomly into the genome of T cells (29).

On the other hand, transposons are a more cost-effective option as they do not need the extensive viral manufacturing process. While they theoretically allow for transgene integration in less critical areas of the genome compared to viruses, potentially making them safer, they still insert genes randomly (161–163). In 2016, the first clinical trial using a non-viral Sleepy Beauty system to generate anti-CD19 CART cells was published (164). This method is not as established as the viral technology and requires further research (165).

Finally, mRNA transfer through electroporation is highly efficient in terms of cost, time, and achieving desired expression levels. However, CAR expression will decrease and eventually fade away in days or weeks as T cells divide, since RNA is not integrated into the genome (139, 166). A CART product that used mRNA electroporation to transiently express the CAR was found to be safe but did not show efficacy in AML patients (75).

To date, most CART cells used to treat AML patients have been genetically engineered using retroviral (108, 151, 167) or lentiviral methods (83, 101, 154). This approach allows for efficient and reproducible genetic modification of T cells that can be produced at scale following good manufacturing practices (GMP).

### 3.4 *In vitro* efficacy testing

Typically, confirmation of CAR expression on T cells is determined using either by flow cytometry or real-time PCR (168). Additionally, the impact of CAR expression on T-cell subsets distribution and the expression of exhaustion and senescence markers can be assessed using flow cytometry. Subsequently, *in vitro* efficacy is evaluated by testing: 1) cytotoxicity towards tumour cells, 2) proliferation and 3) cytokine production (169).

Cytotoxicity is studied by co-culturing CART cells with AML target cells at various effector: target ratios (ranging from 10:1 to 1:8) for different durations (from 4 to 96 hours). The commonly utilized AML models include the MOLM-13/14, THP-1, Kasumi-1 and K562 cell lines. The latter is derived from a chronic myeloid leukemia at a blast crisis. It is crucial to determine the level of expression of the target antigen in the chosen cell line(s). Using one or more cell lines with varying expression levels (high/low) can be advantageous. Target cells can be easily distinguished from CART cells through flow cytometry by transfecting them with a reporter like green fluorescent protein (GFP) or a monomeric cherry red fluorescent protein (mCherry) (170). Common cytotoxicity assays are the 4-hour Chromium-51 (^51^Cr) release assay (108, 171); the luciferase killing assay (172); and assays where the surviving target cells are quantified by flow cytometry at the end of the co-culture (e.g., with 7-AAD or GFP/mCherry as preciously explained) (101). Based on the existing literature, it is anticipated that effective CART cells can eliminate the majority of target AML cells after 24 or 48 hours at low effector: target ratios (101).

Efficient CART cells demonstrate the ability to multiply when they engage with their specific antigen. This can be evaluated by co-culturing previously labelled CART cells (using carboxyfluorescein succinimidyl ester (CFSE)) with one or more target cells, and then tracking their proliferation by measuring CFSE dilution over several generations using flow cytometry (112). In this assay, IL-2 can be used as a positive, non-specific stimulus.

Additionally, when in contact with target cells, CART cells rapidly produce and release various cytokines. In most studies, this is determined by co-culturing CART cells with target cells that either express or do not express the specific antigen (e.g., CD123-directed CART cells are co-cultured with CD123+ and CD123- AML cells). The levels of these cytokines in the culture medium are typically measured using enzyme-linked immunosorbent assay (e.g. ELISA) (171). Specifically, IL-2 is measured to assess the activation status of CART cells, while interferon γ (IFN-γ) and granzyme B are checked to evaluate their cytotoxic activity. Both aspects can also be evaluated using CD107a degranulation assay; CD107a expression indicates the activation and cytotoxic degranulation state of immune cells (173). CART cells also release numerous pro-inflammatory cytokines, such as tumour necrosis factor a (TNF-α), interleukin 6 (IL-6), and interleukin 1b (IL-1b), which play a key role in activating the immune system. However, an exacerbated secretion of these molecules may lead to a strong cytokine release syndrome (CRS), although this can be managed with treatments like anti-IL-6 antibodies (174).

### 3.5 *In vitro* safety assessments

Considering the characteristics of most AML antigens, it is crucial to investigate whether CART cells can recognize the target antigen on HSC and assess if they might eliminate or hinder the proliferation of these cells, potentially causing haematologic toxicity.

To carry out these tests, HSC may be obtained from a cord blood unit or from the bone marrow (BM) of a healthy donor for allo-HSCT (151). These samples can then be used to measure the expression of the target antigen using flow cytometry. If the target antigen is present, cytotoxicity assays and proliferation colony-formation unit assays can be performed after co-culturing the HSC with CART cells. This approach has been employed to establish that both CD123- and CD33-directed CART cells reduced the capacity of HSC to form colonies (101, 167), suggesting that targeting these antigens may lead to myelosuppression. Indeed, in a clinical setting, it is essential to have an available allogeneic donor in case patients require an allo-HSCT after CD123- or CD33- directed CART treatment.

### 3.6 *In vivo* efficacy studies

To assess the efficacy of a novel CART cell in a living organism *in vivo*, it is recommended to initially study their ability to eliminate grafts of AML cell lines first, followed by assessments on AML patient-derived xenograft (PDXs). Nod-scid-gamma (NSG) mice are typically the preferred model for *in vivo* experiments (175) due to their immunodeficiency, which allows human AML cells to engraft in their bone marrow. To improve cell engraftment of cells, especially that of PDX cells, mice are often sub-lethally irradiated 4 to 24 hours prior to the injection of AML cells. The majority of published *in vivo* studies employ AML cell lines like MOLM-13 (101), MOLM-14 (166), THP-1 (176), or Kasumi-1 (175). In these cases, leukemic cells are usually modified with a plasmid expressing the luciferase enzyme, allowing their growth to be periodically monitored through bioluminescence imaging after injecting luciferin intraperitoneally in live mice. Additionally, cells can be transfected with a reporter, such as GFP, to assess their presence in the peripheral blood during the experiment and in other tissues at the end of the study using flow cytometry (139, 167).

At the beginning of the experiment, AML cells (typically 0.1-10x10^6^) are intravenously (i.v.) injected into mice. Once the AML is established, it can be detected through bioluminescence. Following this, which can take from one to several days, CART cells can be i.v. injected into the mice. The dosage of AML cells and CART cells will be determined by the AML proliferation rate and the specific objectives of the study. The most important measurement in these experiments is to quantify the presence of malignant cells.

### 3.7 *In vivo* safety studies

For investigating potential myelotoxicity induced by CART therapy *in vivo*, utilizing a humanized mouse model, which involves mice with a “human” immune system, is the most effective approach. In this model, a human immune system is established by i.v. injecting human HSC or PBMC (175, 176) into previously irradiated NSG or NSG-SGM3 mice (6-12 weeks old). The NSG-SGM3 strain, while more immunosuppressed than NSG, allows for superior engraftment of human cells, leading to *in vivo* expansion of a greater number of human cells from both lymphoid and myeloid lineages. It is worth noting that this strain is more expensive and delicate.

**Figure 5:**
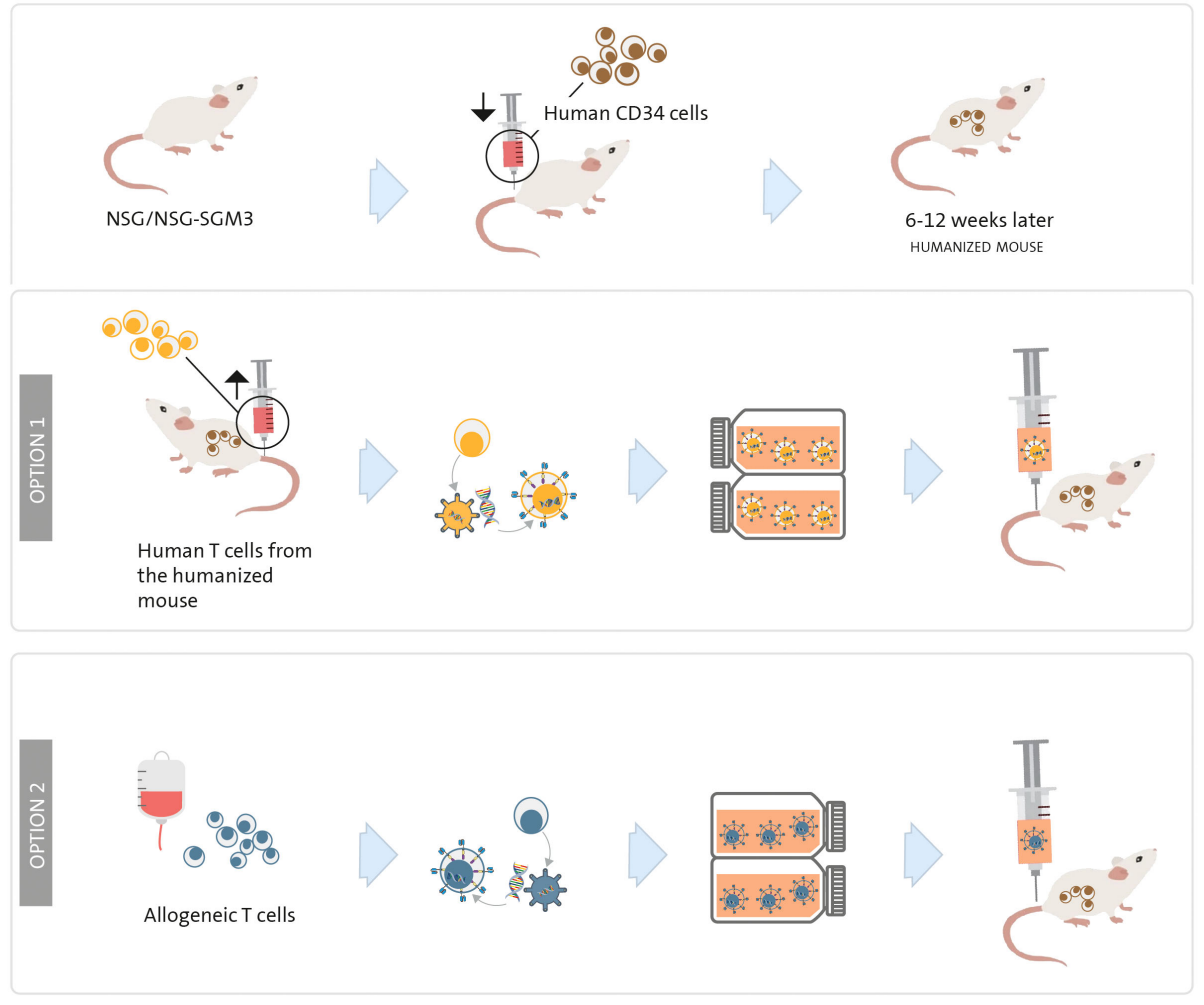
Humanized mouse model to study myelosuppression. NSG or NSG-SGM3 mice are irradiated on day 0 and a human immune system is established by human CD34^+^ haematopoietic stem cells or PBMC transplantation. Once the animals present a “human” immune system (between 6 and 12 weeks later), human CART cells are injected; they can be engineered with T cells obtained from the same humanized mouse (option 1) or from a human T-cell donor (option 2).

Once the haematopoietic system is established (approximately 6 to 12 weeks after HSC or PBMC injection), animals are treated with either autologous human CART cells (derived from the humanized mice) (**Figure 5** option 1) or heterologous CART cells (derived from a human donor) (**Figure 5** option 2). In the latter case, it is important to consider a potential allogeneic effect if the HSC/PBMC and T cells are sourced from different donors (and are not HLA-matched). To evaluate the myelotoxicity induced by CART cells, the condition of the animals and the persistence of HSC will be regularly monitored. The persistence of HSC can be assessed during the experiment through bone marrow aspirates and in peripheral blood, or at the end of the experiment through flow cytometry quantification of HSC in the bone marrow (typically 4 to 12 weeks after CART injection) (120, 177).

The humanized mouse model can also be utilized to evaluate efficacy. This can be achieved by injecting HSC into the liver of sub-lethally irradiated newborn NSG-SGM3 mice (176) or by following the method described in the previous paragraph (141); waiting until the animals develop a human immune system; injecting AML cells as previously outlined; and finally, administering CART cells engineered from T cells obtained from the humanized mice. This model enables simultaneous study of both myelotoxicity and efficacy (elimination of AML cells).

Additionally, a humanized mouse model proves valuable in to assessing CRS potentially caused by the CART cells. These animals contain human monocytes, T cells, and other cells implicated in this syndrome (176).

## 4 Translation of CART therapies for AML

### 4.1 GMP production of CART therapies

At the Hospital Clínic of Barcelona, we have developed several academic CART products, including varnimcabtagene autoleucel (ARI-0001) for patients with R/R B-ALL, B-cell lymphoma and CLL, as well as cesnicabtagene autoleucel (ARI-0002h) for R/R MM patients (21, 169, 170). Drawing from our experience in conducting clinical trials with CART cells and insights from existing literature, we will now outline the requirements for GMP production of CART therapies.

As previously mentioned, in the majority of clinical trials involving CART therapies for AML, autologous T cells are preferred over allogeneic T cells. Autologous T cells are obtained via leukapheresis from the patient, followed by a T-cell selection process (**Figure 1**). After 24 h, retroviral or lentiviral vectors that were previously manufactured are introduced to the cell culture in order to transduce T cells in accordance with GMP standards. Subsequently, the CART cells need to be expanded for a period ranging from 6 to 12 days (depending on required dosage for each clinical trial. This is done in specialized, close-automated bioreactors such as the CliniMacs Prodigy® (178), Wave® Bioreactor (154), G-Rex® or Cocoon® (179).

Of note, Milone’s group achieved highly effective lentiviral transduction of non-activated T cells, managing to produce CART cells within 24 hours (180). Both the CliniMacs Prodigy® and the Cocoon® offer complete automation of the entire production process, ensuring sterility and enhancing reproducibility under GMP conditions. In our view, these bioreactors currently represent the best options for point-of-care production of CART cells (181). CART cells are typically cryopreserved while undergoing sterility and microbiological safety assessments. They are thawed just prior to infusion into the patient. In certain clinical trials (e.g., Atalanta-1/CP0201-NHL), fresh CART cells are administered to patients with a vein-to-vein time of only 7 days. In such cases, a sample is rigorously assessed for safety beforehand.

CART cells must meet specific criteria during the final stages of the manufacturing process. These criteria encompass aspects such as visual appearance, viability, quantity, potency, sterility and safety. Only after meeting these standards can the CART cells be approved for release.

To gain regulatory approval for a clinical trial, the validation of the CART production process under GMP must be submitted to the national regulatory agency. This validation process meticulously covers every aspect of the CART therapy production, ensuring compliance with GMP and conducting through quality controls to ensure safety and evaluate efficacy (168).

### 4.2 Monitoring patients treated with CART cells

The emergence of CART therapies for haematologic malignancies requires specialized training for healthcare professionals, including physicians and nurses. Additionally, it entails the establishment of specific units dedicated to the administration and management of these treatments.

Clinical trials involving CART therapies for AML entail the collaboration of various hospital departments. These include: 1. Haemato-Oncology Department: responsible for treating the patient and, if necessary, overseeing the allo-HSCT; 2. Apheresis Unit: where T cells are collected from the patient; 3. Immunotherapy Unit: this Unit is responsible for generating or receiving, processing and analysing the CART product; and 4. Intensive Care Unit (ICU) that may also be involved in patient care (21).

Serious side effects, such as CRS and neurological toxicity, have been reported in up to one third of AML patients undergoing CART therapies (182, 183). Other reported adverse effects include infections, persistent cytopenia, macrophage activation syndrome, as well as various on-target/off-tumour effects.

Cytokine-release syndrome results from the massive release of inflammatory cytokines (e.g., IL-1 and IL-6) following target recognition by the CART cells. This leads to the activation of other immune cell, like tissue macrophages, which can induce changes or damage in extra tumoral tissues. Neurotoxicity, also known as immune effector cell-associated neurotoxicity syndrome (ICANS), shares a similar underlying pathophysiology with CRS. In this case, the inflammatory cytokines produced by CART cells and the tumour microenvironment diffuse into the central nervous system. This, in turn, can trigger activation of microglial cells, leading to neurological symptoms (184).

As previously mentioned, the majority of the antigens found on AML cells are also present on healthy HSC. CART cells targeting these antigens could potentially result in prolonged cytopenia. Specialized care is required to manage these conditions. Therefore, utilizing risk-stratification tools like the CAR-HEMATOTOX score may prove beneficial in assessing the risk of hematotoxicity and anticipating patient needs. This tool can correlate the duration of severe neutropenia based on predictive biomarkers of hematotoxicity for R/R B-cell lymphoma (185). In 2023, a comprehensive survey conducted by EHA-EBMT groups outlined grading and management guidelines for hematoxicity following CART therapy. The results emphasized the widespread use of CTCAE (Common Terminology Criteria for Adverse Events) criteria for grading post-CART cytopenias and CIBMTR for evaluating hematopoieitic recovery by clinicians (6).

Given the anticipated side effects, AML patients who are considered for CART therapy must exhibit a satisfactory performance status and lack major comorbidities that could potentially worsen their prognosis. Eligibility assessments should be conducted by a multidisciplinary team experienced in this form of therapy, and equipped with the necessary resources to manage or treat potential complications.

### 4.3 Clinical trials utilizing CART cells in AML

The inaugural Phase I trial evaluating CART cells in AML patients was documented in 2013. This trial utilized a second-generation CAR featuring a CD28 costimulatory domain, targeting the LeY antigen. Four patients with R/R AML participated (74), and this trial yielded the initial indication of CART activity against AML in humans. Notably, three patients exhibited either disease stability or a reduction in blast count. Moreover, one patient with skin infiltration (known as *leukemia cutis*) experienced a transient improvement, as confirmed by a lymphocytic infiltration in the skin biopsy. This finding suggested that CART cells may be effective in eliminating AML in cases of extramedullary disease.

**Figure 6:**
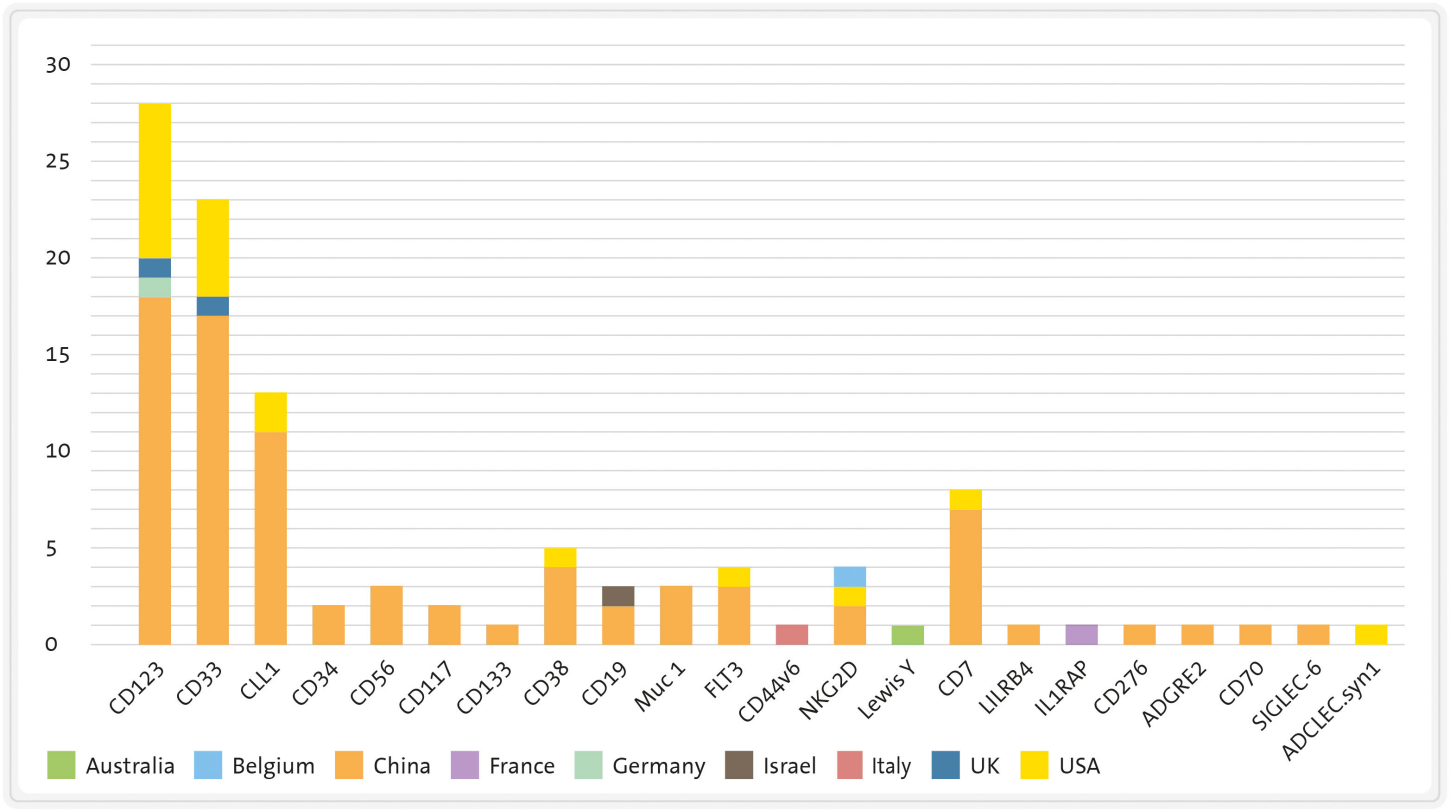
Number of clinical trials of CART therapies for AML divided by target antigen and country. CLL1, C-type lectin-like molecule-1 FLT3: FMS-like tyrosine kinase 3 LILRB4, leukocyte immunoglobulin like receptor B4. Source: www.clinicaltrials.gov (26/06/2023).

Currently, CD123 is the most frequently targeted antigen by CART therapies in AML patients (see **Figure 6**). As mentioned earlier, CD123 is expressed in the majority of AML cases and its expression has also been identified on LSC (177, 186). However, it is also present in endothelial cells, which means that on-target/off-tumour toxicity could lead to life-threatening consequences, such as capillary-leak syndrome (103). Moreover, since CD123 is found in HSC, CD123-targeted CART cells may impair normal haematopoiesis and potentially cause irreversible myeloablation, though this is still a subject of debate (100, 101, 187). The first patient treated with an allogeneic “universal” CD123-directed CART therapy (UCART123; NCT03203369) was a 78-year-old male diagnosed with BPDCN who died due to a combination of CRS and capillary-leak syndrome. Consequently, the trial was initially discontinued (188) and later resumed with a dose reduction and the introduction of an upper age limit (78).

Another trial employed a distinct strategy to decrease toxicity: a “biodegradable” CD123-directed CAR through mRNA electroporation (75). Published data revealed that all treated patients experienced fever, and four out of five suffered from CRS; the trial was discontinued due to lack of antitumoral efficacy.

Finally, ongoing trials in the USA and China (NCT02159495, NCT04318678, and NCT03114670) involve a CD123 CAR construct that also expresses EGFR or CD20 on the T-cell surface, providing a safety switch off upon treatment with EGFR/CD20-specific antibodies (cetuximab and rituximab, respectively) (77, 145).

CD33 is another target currently being explored in clinical trials for AML patients (80, 167, 186). However, there have been limited published results from registered trials involving CART (NCT01864902, NCT0186902, and NCT03126864) or CAR-NK cell therapies targeting CD33 (NCT02944162) in AML. Preliminary findings from a trial conducted in China (NCT01864902) indicated that one patient experienced an initial reduction in blasts and systemic inflammatory symptoms; however, after two weeks, the disease progressed (79). Notably, this patient did not exhibit clinical or analytical signs of sinusoidal obstruction syndrome, a complication associated with GO, as previously described (189).

A CART therapy targeting two antigens, either CD33 or CLL-1, is currently undergoing phase I trial testing (NCT03795779). It has been reported that a patient enrolled in this study was able to undergo allo-HSCT after successful eradication of leukemia and myeloablation (81).

The most recent clinical trial utilizing CART therapy directed specifically towards CLL-1, either alone or in combination with CD33 as previously mentioned (81), obtained promising outcomes in both adult patients (84) and paediatric R/R AML patients (82, 85), (**Table 1**). Among the eight paediatric patients treated, four achieved a state of morphologic leukemia-free state (MLFS) along minimal residual disease (MRD) negativity (85).

A CART therapy targeting NKG2D has also undergone evaluation in a phase I trial encompassing AML and other haematological neoplasms (NCT02203825) (86). In this study, CART cells exhibited biological activity, as evidenced by an increase in inflammation parameters post-injection. Nevertheless, their anti-AML effect was limited (190). The potential improvement of clinical efficacy through combination with therapies that upregulate NKG2D expression, such as decitabine (191), warrants further investigation.

Furthermore, CART therapies targeting additional antigens are currently undergoing patient trials, albeit with limited published data, as seen in trials NCT03473457, NCT03291444, NCT04351022, etc. See **Tables 1** and **2** for detailed results of published clinical trials for AML (87, 88).

**Table 2:** Published clinical trials using CART in AML: Clinical results.

Clinical trial number	Phase	Lymphodepletion	Dose	Indication	Patients	Response	Toxicity	Country	Reference
NCT01716364	I	F-Cytarabine	1.1x10^9^ (range 5 × 10^8^/kg to 1.3 × 10^9^/kg)	AML	4	2 SD; 1 transient blast reduction; 1 transient cytogenetics remission	CRS: Grade I/ II (neurotoxicity no comments)	Australia	(74)
NCT02623582	I	Optional C	Cohort 1: 3 doses, each 4x10^6^ cells/kg; Cohort 2: 6 doses, each 4x10^6^ cells/kg	AML	7 (5)	5 PD	CRS Grade I: 8%; grade II: 33% ; grade III: 50%; grade IV: 8%; No vascular, neurological or hematologic toxicity	USA	(75)
NCT02159495	I	FC	DL 1: 50x10^6^ CART; DL 2: 200x10^6^ CART	AML/ BPDCN	7 (6)	1 MLFS ; 2CR; 2 blast count reductions	CRS 5 pts grade I-II; 1 rash grade III (neurotoxicity no comments)	USA	(76)
NCT04318678	I	FC	DL1: 3x10^5^/kg, DL2: 1x10^6^/kg, DL3: 3x10^6^/kg, DL4: 1x10^7^/kg	Pediatric AML	12	1 blast count reduction; 1 CR.	CRS Grade I; no neurotoxicity	USA	(77)
NCT04106076	I	FC/ FCA	DL1: 2.5x10^5^; DL2: 6.25x10^5^; DL2i: 1.5x10^6^; DL3: 3.03x10^6^	AML	16	4 pts: 2 SD; 1 MLFS; 1 MRD-negative CR	CRS: 15 pts (3 pts ≥ grade III; (2: grade III; 1 grade V)) ICANS: 1 pt≥ grade III	USA	(78)
NCT01864902	I/II	No	1.12x10^9^ ; fraccionated doses on consecutive days: 1 × 10^8^, 1.2 × 10^8^, 4 × 10^8^, 5 × 10^8^	AML	1	Blast count reduction	CRS grade IV, pancytopenia	China	(79)
NCT03927261	I	No FC/ FC	1.8-83 x10^6^ CART cells	AML/ CMML/ MDS	24 (20)	7 Blast count reduction; 1 CRi; 1 CR; 1 PR; 1 SD	CRS grade I: 10 pts; grade II: 6 pts; grade III: 1 pt. No cases of bone marrow aplasia	USA	(80)
NCT03795779	I	FC	Two doses, each of 1x10^6^ CART/kg on consecutive days	AML	NA	1 CR	NA	China	(81)
NA	I	NA	0.35-1x10^6^CART/kg	Peadiatric AML	3	2 MRD- CR; 1 MRD+ CR	CRS: grade I: 2 pts; grade II: 1 pt; no neurotoxicity; pancytopenia	China	(82)
NCT03222674/Pediatric ChiCTR1800015883	I/II	FC	1x10^6^ CART/kg	Pediatric AML	4	3 CR; 1 PD	CRS:grade I: 1pt; grade II: 2 pts ICANS: 1pt grade I/II, no HLH, transient grade IV neutropenia	China	(83)
ChiCTR2000041054	I	FC	1-2x10^6^ CART/kg	AML	10	3 MRD+ CR/CRi; 4 MRD- CR/CRi; 3 PD	CRS grade I-II: 4 pts; grade III-IV: 6 pts; severe pancytopenia: 10 pts (incl. 2 deaths due to severe infection), no neurotoxicity	China	(84)
NCT03222674/Pediatric ChiCTR1900027684	I/II	FC	0.35- 1x10^6^/kg CART	Pediatric AML	8	4 CR MRD-; 1 CRi MRD+; 1 MLFS MRD+; 1 PR; 1 SD	CRS: grade I-II: 8 pts; no neurotoxicity; 8 pancytopenia	China	(85)
NCT02203825	I	No	DL1: 1x10^6^; DL2: 3x10^6^; DL3: 1x10^7^; DL4: 3x10^7^ (T cells)	AML/MM	12 (7)	2 SD; 5 PD	No CRS, noneurotoxicity, no autoimmunity, 2/12 grade 1 rash	USA	(86)
NCT04351022	I/II	FC	6.1-10x10^6^/kg	AML	6	1 CR, 3 CRi, 1 blast count reduction, 1 PD	CRS: grade I-II: 5 pts; grade III: 1 pt; no neurotoxicity,no GvHD, neutropenia (<500/mcL) 6 pts, thrombocytopenia (<10000/mcL) 6pts	China	(87)
NCT04538599	I	FCE	1-3x10^7^/kg	CD7-positive haematological malignancies (1 AML)	12 (1)	1 CRi	CRS: grade I, no neurotoxicity, no GvHD	China	(88)

Only data from AML patients are reported. Cell dose is reported for all patients included in the clinical trial. Total cell dose indicated, if not otherwise specified (i.e. CART/kg). Total number of treated patients in the clinical trial and AML treated patients in parenthesis. Toxicity is reported about AML patients or all patient if is not specify. A, alemtuzumab; BPDCN, blastic plasmacytoid dendritic cell neoplasm; C, Cyclophosphamide; CR, complete response; CRi, complete response with incomplete haematological recovery; CRS, cytokine release syndrome; E, etoposide; F, Fludarabine; HLH, hemophagocytic; HMA, hypomethylating agent; MDS, myelodysplastic syndrome; MLFS, morphologic leukemia-free state; MM, multiple myeloma; MRD, measurable residual disease; NA, not available; PD, progression disease; PR, partial response; SD, stable disease.

### 4.4 Combining CART therapy and allogeneic HSCT: a dual immunotherapeutic strategy?

Allogeneic hematopoietic stem cells transplantation remains a cornerstone in the therapeutic plan for eligible AML patients, serving both as a consolidation approach for high-risk AML and as part of a salvage option for R/R AML (141). Therefore, integrating CART therapy into the treatment of these patients needs a comprehensive plan that includes allo-HSCT (192–194).

**Figure 7:**
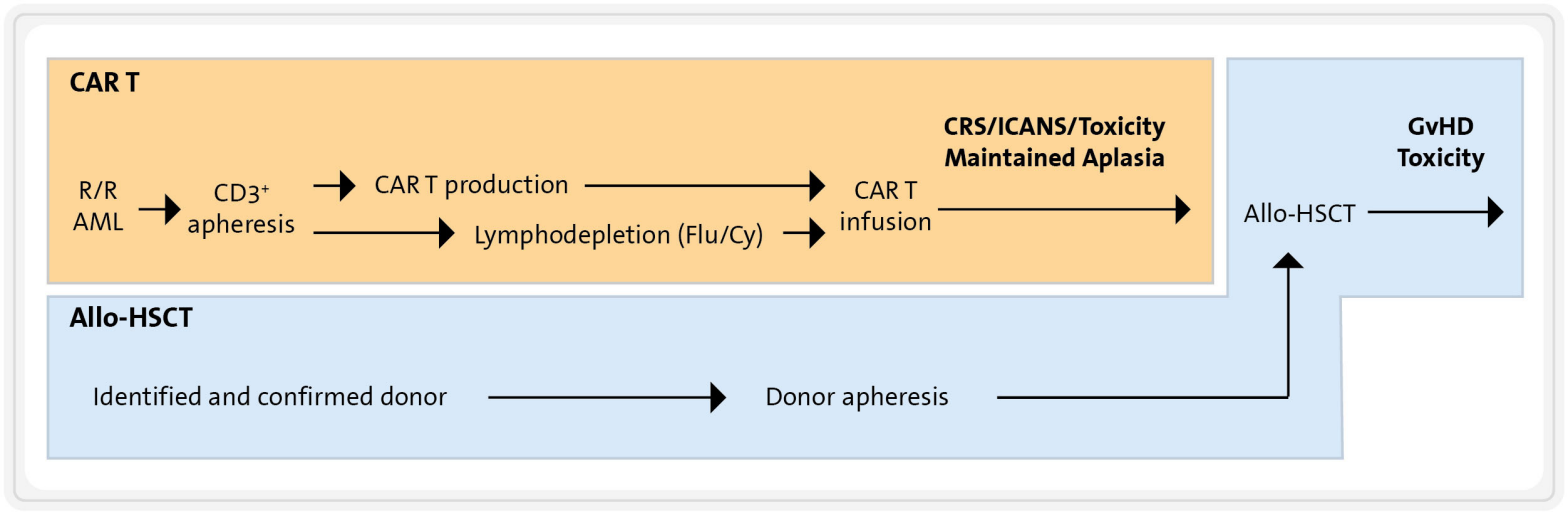
Combination of CART therapy and allo-HSCT. Flu, fludarabine; Cy, cyclophosphamide; CRS, cytokine release syndrome; ICANS, immune effector cell-associated neurotoxicity syndrome; GVHD, graft-versus-host disease.

In the case of R/R AML patients who have not previously undergone allo-HSCT, the administration of a CART therapy followed by allo-HSCT represents a rational strategy. This approach aims to combine the potential benefits of CART infusion, which is critical for achieving AML cytoreduction, with the advantages of allo-HSCT. The latter can provide both additional GvL effects and a haematopoietic rescue after cytopenia probably caused by the CART cells. Executing this combined strategy is nonetheless logistically and clinically challenging, particularly managing the short interval between CART infusion and allo-HSCT (see **Figure 7**). It requires precise coordination with the haematopoietic cell donation process to minimize the period of CART-induced myelotoxicity and maximize the effectiveness of the CART cells. Moreover, the removal of CART cells to avoid interference with engraftment requires the lymphodepleting effects of a conditioning allo-HSCT regimen. Finally, patients may face frequent and sometimes severe complications associated with this sequential tandem procedure. These include immune-mediated complications post-CART infusion (e.g., CRS, ICANS, …) and the complications typically linked with allo-HSCT, such as GvHD and severe immunosuppression.

Furthermore, CART cells hold promise for treating AML who experience a relapse after undergoing allo-HSCT. In this setting, any severe cytopenia following CART infusion could potentially be rescued using the same graft source utilized in the previous allo-HSCT.

## 5 Discussion

While CART therapies have shown remarkable success in treating haematological diseases such as B-ALL, B-cell lymphoma and MM, their application in AML patients has faced greater challenges. The primary hurdle is the lack of a specific antigen exclusively expressed in AML cells. Despite this, clinical trials have predominantly focused on targets like CD123, CD33 and CLL-1. While published clinical data offer glimpses of the potential of CART therapies in AML treatment, they also underscore the limitations due to on-target/off-tumour toxicities. Therefore, in the majority of clinical trials involving CART therapies for AML, the availability of an allogeneic HSC donor is imperative to eventually rescue patients from life-threatening cytopenia. There are safety strategies, including switch-off mechanisms or the exploration of various dual CAR strategies, that can be employed to mitigate the potential toxicity induced by CART cells in AML (21, 195). Finally, refining clinical protocols, like combining CART cells with other therapies or integrating allo-HSCT, may help unlock the full potential of this therapeutic modality in AML patients, rendering it both safer and more effective (196).

## Author contributions

LP-A: Writing – original draft, Writing – review & editing. ÀB: Writing – original draft. JD: Supervision, Writing – review & editing. JE: Writing – review & editing. MJ: Writing – review & editing, Funding acquisition. NK-G: Writing – original draft, Writing – review & editing, Supervision.
